# RNA-Seq-Based Analysis Reveals Heterogeneity in Mature 16S rRNA 3′ Termini and Extended Anti-Shine-Dalgarno Motifs in Bacterial Species

**DOI:** 10.1534/g3.118.200729

**Published:** 2018-10-24

**Authors:** Jordan R. Silke, Yulong Wei, Xuhua Xia

**Affiliations:** * Ottawa Institute of Systems Biology, Ottawa, Ontario, Canada K1H 8M5

**Keywords:** Gene Expression, RNA-Seq, Translation Initiation

## Abstract

We present an RNA-Seq based approach to map 3′ end sequences of mature 16S rRNA (3′ TAIL) in bacteria with single-base specificity. Our results show that 3′ TAILs are heterogeneous among species; they contain the core CCUCC anti-Shine-Dalgarno motif, but vary in downstream lengths. Importantly, our findings rectify the mis-annotated 16S rRNAs in 11 out of 13 bacterial species studied herein (covering Cyanobacteria, Deinococcus-Thermus, Firmicutes, Proteobacteria, Tenericutes, and Spirochaetes). Furthermore, our results show that species-specific 3′ TAIL boundaries are retained due to their high complementarity with preferred Shine-Dalgarno sequences, suggesting that 3′ TAIL bases downstream of the canonical CCUCC motif play a more important role in translation initiation than previously reported.

Understanding bacterial translation is important to pharmaceutical industries seeking to optimize protein biosynthesis (Xia 2018a). In this process, the rate-limiting step is generally considered to be initiation (Kudla *et al.* 2009; Tuller *et al.* 2010; Xia 2015) and the most prominently cited mechanism of initiation in bacteria (Shine and Dalgarno 1974, 1975) involves an interaction between a pyrimidine-rich anti-Shine-Dalgarno (aSD) sequence at the 3′ end of the 16S rRNA (3′ TAIL) and a purine-rich Shine-Dalgarno (SD) sequence in the mRNA translation initiation region (TIR) of protein coding genes. Pairing between these two sequences helps the ribosome dock near the start codon.

Efficient SD-mediated translation initiation requires optimal SD:aSD binding location and pairing potential (Schurr *et al.* 1993; Osterman *et al.* 2013; Prabhakaran *et al.* 2015; Abolbaghaei *et al.* 2017; Hockenberry *et al.* 2017; Wei *et al.* 2017). The canonical core aSD motif, CCUCC, is widely believed to elevate initation efficiency because of its strong complementarity with SD sequences and conservation across phyla (Shine and Dalgarno 1974; Woese *et al.* 1975; Schurr *et al.* 1993; Starmer *et al.* 2006; Vimberg *et al.* 2007; Nakagawa *et al.* 2010). Yet what constitutes ideal SD:aSD complementarity remains a subject of debate. Some researchers contend that there is weak association between SD:aSD binding affinity and initiation efficiency (Li *et al.* 2012), but others suggest that intermediate binding affinities optimize initiation efficiency in *Escherichia coli* and *Bacillus subtilis* when a broader range of SD:aSD interactions is considered (Vimberg *et al.* 2007; Osterman *et al.* 2013; Hockenberry *et al.* 2017). Furthermore, when a SD sequence that binds to the *B. subtilis* 3′ TAIL is substituted with a shorter SD sequence pairing with *E. coli*’s 3′ TAIL, interferon plasmids’ expression levels decrease drastically (Band and Henner 1984). These findings emphasize the importance of characterizing the full extent of the 3′ TAIL.

The 3′ TAIL boundary remains ambiguous for most bacterial species because the precise 3′ maturation process of the 16S precursor sequence remains unclear (Sulthana and Deutscher 2013; Deutscher 2015), and only a few mature 16S rRNA sequences have been experimentally verified (Woese *et al.* 1980). Consequently, determination of the 16S rRNA is frequently automated based on sequence similarity (Lin *et al.* 2008; Nakagawa *et al.* 2010). However, this process is often unreliable (Starmer *et al.* 2006; Jones *et al.* 2007; Lagesen *et al.* 2007; Lin *et al.* 2008) and many such 16S ribosomal RNA sequence annotations have been discontinued in NCBI’s Gene database. For example, 16S rRNA entries for *Streptococcus pyogenes* (NC_002737), *Bacillus anthracis* (NC_005945), and *Legionella pneumophila* (NC_005823) are all truncated such that their annotated 3′ ends do not encompass the canonical CCUCC motif.

To circumvent the aforementioned problem, we devise strategies to map RNA transcripts from high-throughput RNA sequencing (RNA-Seq) data (Lister *et al.* 2008; Wang *et al.* 2009; Anders *et al.* 2013) to the 16S rDNA genomic sequence with single base specificity. The feasibility of this approach was shown recently in a study (Wei *et al.* 2017) where we successfully recovered the *E. coli* and *B. subtilis* 3′ TAILs documented in literature (Shine and Dalgarno 1974; Woese *et al.* 1975). Our present objective is to advance our RNA-Seq framework to characterize the 3′ TAIL in any bacterial species, especially those that have not been experimentally verified.

The challenge associated with our approach is the limited availability of suitable data. There is a complete lack of publicly available RNA-Seq data in GEO DataSets for many species, such as *Acidithiobacillus ferrooxidans*, *Microcystis aeruginosa*, *Shigella flexneri*, and *Yersinia pestis*. Furthermore, many experiments remove rRNAs prior to sequencing (O’Neil *et al.* 2013) in an effort to enrich the target RNA molecules, such as mRNAs (Choi and Hagedorn 2003). Fortunately, our findings suggest that ribo-depletion is often incomplete, and enough 16S rRNA reads will persist to allow for 3′ TAIL characterization. The inclusion of 13 species studied herein (covering Cyanobacteria, Deinococcus-Thermus, Firmicutes, Proteobacteria, Tenericutes, and Spirochaetes) is thus predicated on the availability of usable RNA-Seq datasets in NCBI’s GEO (Edgar *et al.* 2002) database (see Materials and Methods for additional details). Additionally, the availability of protein abundance data in PaxDb (Wang *et al.* 2012, 2015) for all species studied allow us to investigate the effect of SD:aSD complementarity on protein production in real genes.

Comprehensive comparative sequence analyses (Nakagawa *et al.* 2010, 2017) claim 5′-CCUCCU-3′ is the functionally constrained 3′ TAIL terminus. In other words, the motif is conserved among bacterial species because it pairs with SD sequences effectively. However, several bases further downstream are conserved in the genomic sequences of closely related species. We suspect that this is the result of functional constraint imposed by the SD:aSD interaction further downstream of 5′-CCUCCU-3′. Accordingly, we hypothesize that downstream bases are retained in 3′ TAILs because they effectively interact with species-specific SD sequences as previously observed for *E. coli* and *B. subtilis* (Band and Henner 1984; Abolbaghaei *et al.* 2017; Wei *et al.* 2017).

Our findings corroborate previous studies suggesting that intermediate binding affinity is preferred (Osterman *et al.* 2013; Hockenberry *et al.* 2017; Wei *et al.* 2017). The 3′ termini downstream of the core CCUCC are heterogeneous among species, but fall within the conserved boundary at the genomic level. Furthermore, terminal bases are preferred in SD:aSD binding in most species, albeit having weaker binding affinity than CCUCC. These findings demonstrate the importance of considering bases downstream of CCUCC in SD:aSD binding.

## Materials and Methods

### Processing genomic and RNA-Seq data

The annotated genomes of 26 species in GenBank formats were retrieved from the National Center for Biotechnology Information (NCBI) database (http://www.ncbi.nlm.nih.gov). Next, the NCBI annotated 16S rRNA was retrieved. In the case where multiple 16S rRNA entries exist, the first one listed is selected.

**Figure 1:**
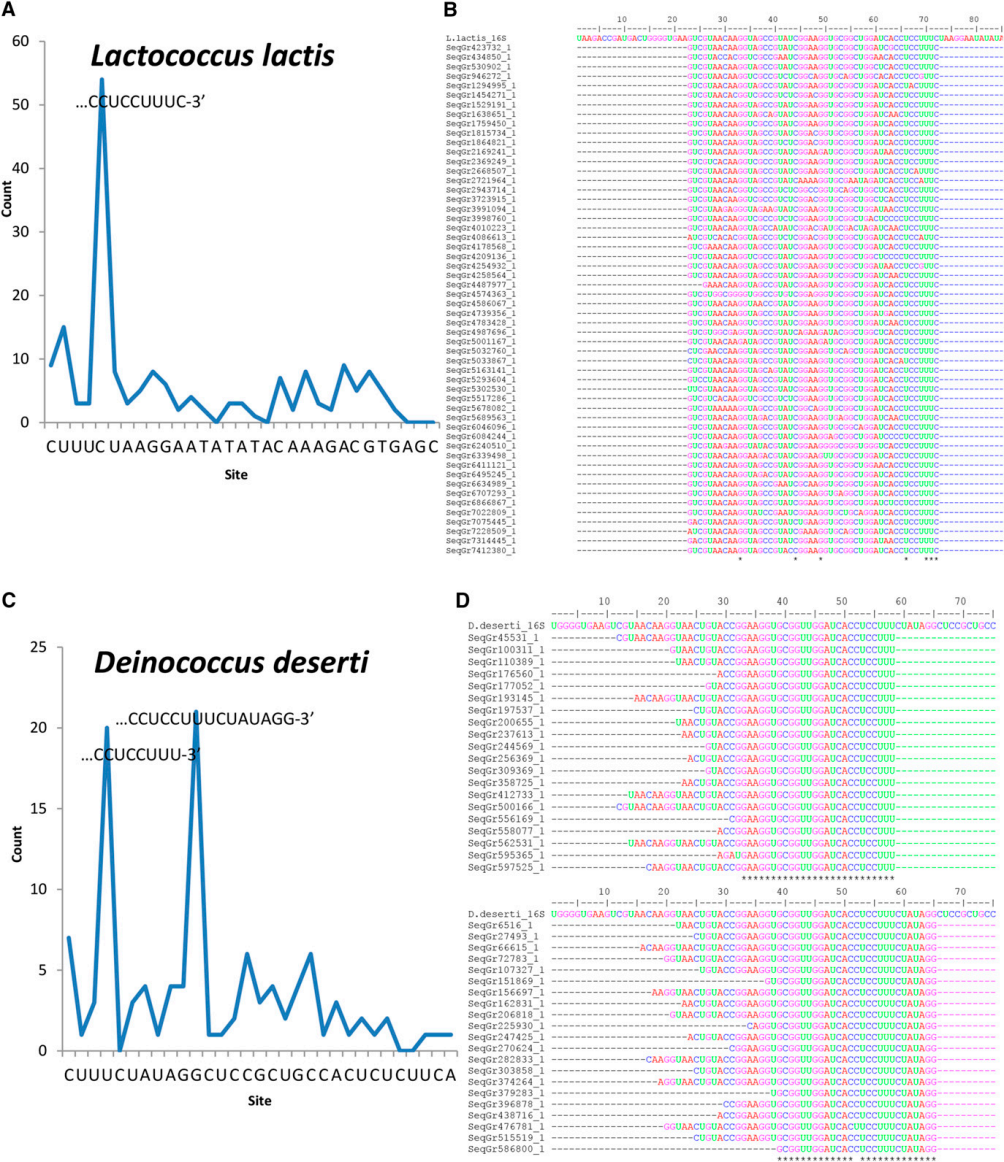
The count of mapped 3′ ends of RNA-Seq reads (A, C) and sequence alignments (B, D) for *Lactococcus lactis* and *Deinococcus deserti*. Mapped regions start with the last C of CCUCC as the first site, extended by 30 nt downstream. The 3′ ends of sequence alignments represent local reads mapped to the single major peak in *L. lactis* and the two major peaks in *D. deserti*. The complete length of the query genomic sequence is 205 nt long.

High-throughput RNA-Seq SRX runs of wildtype species were downloaded from GEO DataSets in FASTQ format. The FASTQ files were first converted to FASTQ+ format using ARSDA 1.1 (Xia 2017), grouping identical reads under a single ID while also indicating the number of copies (SeqID_# of copies), in order to reduce the size of the datasets prior to adapter trimming. The FASTQ+ data were then processed using CutAdapt 1.17 (Martin 2011) to trim off the 3′ flanking adapter sequences. In experiments that use the oligo(dT)-adapter primer, RNA fragments are first poly-adenylated at the 3′ end, we thus set CutAdapt to recognize “AAAAA”. In others that use specific sets of primers ligated to random hexamers, we set CutAdapt to recognize all possible adapters in the kits’ index, with 10% error rate. Regardless of whether poly-As or barcode adapters were trimmed, we only retained reads that were 25 nt or longer after the trimming process to mitigate bias in expression levels (Williams *et al.* 2016). Next, we used Trimmomatic 0.38 (Bolger *et al.* 2014) to remove poor quality sequences with average Phred scores lower than 20 (1% probability of a base calling error) (Ewing and Green 1998). Since adapters were trimmed after reads were grouped in FASTQ+ format, sequences that were previously unique due to the presence of adapter nucleotides may become identical (such as for SeqGr176560_1 and SeqGr558077_1, Figure 1d). The processed FASTQ+ datasets were subsequently converted into FASTA format for multiple sequence alignment.

### Aligning RNA-Seq reads to annotated rRNA sequences

We next mapped reads in the FASTA files onto the 16S rDNA genomic sequence. The FASTA+ files were converted into BLAST databases using the “Create BLAST DB” function in ARSDA. The BLAST query sequence was selected using genomic sequences 100 nt upstream and downstream of the core CCUCC motif (205 nt total query length). For each species, the query sequence was searched against BLAST databases using the BLAST function (Altschul *et al.* 1990) implemented in ARSDA. We used an E-value cutoff of 10^−5^ (with the exception of *Bacillus anthracis*, for which we used an E-value of 10^−3^ due to the relatively shorter average read length and smaller database size) paired with a minimum word length of 12 to balance the quantity and quality of hits, as well as search speed, against the >= 25 nt reads in the ribo-depleted datasets. Then, sequence hits were retrieved from the FASTA files using seqtk (Li 2012) and complementary strand sequences were eliminated. Finally, remaining hits were aligned to the query sequence using multiple sequence alignment (Clustal Omega algorithm (Sievers and Higgins 2014) implemented in DAMBE, default parameters).

### Determining putative SD sequences based on pairing potential, location, and binding affinity

For each species, our characterized 3′ TAILs (Table 1) were used as the complementary sequence in identifying putative SD sequences. To ensure that determined putative SD sequences are from real genes, we map protein IDs in PaxDb 4.0 (Wang *et al.* 2012, 2015) to Gene IDs in NCBI and only use CDSs that have protein expressions. Using DAMBE7 (Xia 2018b), we followed the methods used in previous studies (Nussinov *et al.* 1978; Waterman and Smith 1978): 30 nt upstream of start codon of all CDSs were extracted and matched against the annotated 3′ TAIL with ‘Analyzing 5′UTR’ in DAMBE, with minimum SD length =4 nt and maximum SD length = 12 nt. Site-specific observed and expected aSD usage values were retrieved from the DAMBE when SD sequences are determined.

**Table 1:** The RNA-Seq corrected 3′ TAIL in 13 bacterial species. RNA-Seq determined 3′ TAILs are shaded gray. The NCBI annotated 3′ TAILs are in black fonts, extensions revealed by RNA-Seq data are underlined and ambiguities in bold

Species	16S 3′ TAIL	Putative pre-16S rRNA	NCBI accession	SRA accession
***Listeria monocytogenes***	GAUCACCUCCUUUCU		NC_003210	SRX2771238-41
Streptococcus pyogenes*	GAUCACCUCCUUUCU		NC_002737	SRX3036007, 08, 10, 11
Lactococcus lactis*	GAUCACCUCCUUUC		NC_002662	SRX2140913
Bacillus anthracis*	GAUCACCUCC		NC_005945	SRX129739
Neisseria meningitidis*	GAUCACCUCCUUUCU**A**^†^		NC_003112	SRX2005108, 10
Clampylobacter jejuni*	GAUCACCUCCUUUC		NC_002163	SRX326863
Deinococcus deserti*	GAUCACCUCCUUUCUA	GAUCACCUCCUUUCUAUAGG	NC_012526	SRX497284
Mycoplasma pneumoniae*	GAUCACCUCCUUUCUAAUGGAG	GAUCACCUCCUUUCUAAUGGAG	NC_017504	SRX1122953
Salmonella enterica*	GAUCACCUCCUUA		NC_003198	SRX2409112, 3
Legionella pneumophila*	GAUCACCUCC	GAUCACCUCCUUACAUAGAAAGGCAC	NC_002942	SRX041877
Desulfovibrio vulgaris*	GAUCACCUCCUU		NC_002937	SRX066256
***Leptospira interrogans***	GAACACCUCCUUUUUAAGGAG	GAACACCUCCUUUUUAAGGAGAAUCAAAGG	NC_005823	SRX2448245-52
*Synechocystis* sp.*	GAUCACCUCCUUUAAGGG		NC_000911	SRX2694285-8

* Species whose characterized 3′ TAIL differ from NCBI annotation.

† The use of poly-adenylated data makes it difficult to determine whether the terminal nucleotide is U or A in this case.

### Data Availability

Supplementary file S1 contains RNA-Seq BLAST hits and file S2 contains the list of genes with protein abundance data that were used to determine putative SD sequences in all species studied; Figure S1 contain the 3′ TAIL map for the remaining 11 species. Supplemental material available at Figshare: https://doi.org/10.25387/g3.7081094.

## Results and Discussion

### Characterizing the 3′ TAIL in bacteria using an improved RNA-Seq-based approach

We improve upon our method of 3′ TAIL characterization (Wei *et al.* 2017) by processing the RNA-Seq data more rigorously. To ensure quality and single-base specificity for reads mapped to a reference genomic sequence, we used CutAdapt (Martin 2011) to trim adapters flanking raw RNA-Seq reads because these sequences obscure the true end of RNA fragments (see Materials and Methods for more detail). We subsequently filtered out poor quality reads by discarding those with average Phred scores ≤ 20 using Trimmomatic (Bolger *et al.* 2014); in other words, we retained reads with average base-calling error rates of < 1% (Ewing and Green 1998). A caveat of using poly-adenylated RNA-Seq datasets for *Neisseria meningitidis* is that we cannot distinguish between 5′-CCUCCUUUCU-3′ and 5′-CCUCCUUUCUA-3′ as the 3′ TAIL; it is unclear whether the first adenosine is associated with the 3′ TAIL or the poly-A chain (Table 1).

To map the 16S rRNA, we generated a BLAST library using the quality filtered datasets and performed ungapped local similarity search using BLAST (Altschul *et al.* 1990) between RNA-Seq reads and a 205 nt genomic sequence with the canonical CCUCC motif at the center (100 nt extending from each side). We next aligned the BLAST hits by multiple sequence alignment (Clustal Omega algorithm (Sievers and Higgins 2014) implemented in DAMBE (Xia 2018b), default parameters) against the reference genomic sequence. In all species, we define the terminus of the 3′ TAIL using two criteria: 1) it must contain the canonical CCUCC, and 2) it is the most mapped site at or near CCUCC. The underlying assumption for the second criterion is that the mature 16S rRNA is more abundant than precursor transcripts, as is the case in *E. coli* (Cangelosi and Brabant 1997), because precursors are continuously degraded by exoribonucleases (Sulthana and Deutscher 2013).

### The 3′ TAIL termini are heterogeneous but functionally constrained

Following our two criteria, we have characterized the 3′ TAIL in 13 out of 26 species in PaxDb (Table 1). Figure 1 shows the sequence map and alignments for *Lactococcus lactis* and *Deinococcus deserti*. The sequences mapped for the 11 others are present in Supplementary Figure S1. Two others, *E. coli* and *B. subtilis*, were previously determined (Wei *et al.* 2017). The remaining 11 species could not be characterized because of the aforementioned absence of data in four species (*Acidithiobacillus ferrooxidans*, *Microcystis aeruginosa*, *Shigella flexneri*, and *Yersinia pestis*), and because no convincing peaks were observed in the region of interest (up to 30 nt downstream of CCUCC) in the remaining seven species (*Bacterioides thetaiotaomicron*, *Bateonella henselae*, *Helicobacter pylori*, *Mycobacterium tuberculosis*, *Pseudomonas aeruginosa*, *Staphylococcus aureus*, and *Shewanella oneidensis*), likely due to effective ribo-depletion in their RNA-Seq datasets. We considered a peak to be convincing when the counts mapping to that site were at least 3 fold higher than background (counts of any four flanking sites on either side). Importantly, in the characterized 13 species, we made corrections to annotations in eight species (NC_002662 *L. lactis*, NC_002163 *Clamylobacter jejuni*, NC_000911 *Synechocystis* sp., NC_003112 *N. meningitidis*, NC_*012526 D. deserti*, NC_017504 *Mycoplasma pneumoniae*, NC_003198 *Salmonella enterica*, NC_002937 *Desulfovibrio vulgaris*), and redefined the 3′ TAIL in three others (NC_002737 *S. pyogenes*, NC_005945 *B. anthracis*, and NC_002942 *L. pneumophila*) that were certainly mis-annotated due to their failure to incorporate the canonical CCUCC. Resultantly, the annotated 3′ TAILs of only two out of 13 species, NC_003210 *Listeria monocytogenes* and NC_005823 *Leptospira interrogans* were left unchanged. In short, the 3′ TAILs can variably extend up to six bases downstream of CCUCC in the majority of species studied.

The 3′ TAILs vary among species, but bases downstream of the CCUCC motif are conserved among bacteria. The 16S genomic sequences are largely conserved for several bases beyond CCUCC: *e.g.*, 5′-GAUCACCUCCUUUCUA-3′ in Bacilli and 5′-GAUCACCUCCUUA-3′ in Beta- and Gammaproteobacteria. This conservation suggests that the regions downstream of CCUCC may also be important in SD:aSD pairing. Importantly, 3′ TAIL terminal bases downstream of CCUCC are species-specific, but do not extend past the conserved genomic boundaries, in all species studied except in *L. interrogans*. In other words, the 3′ TAIL falls variably short of 5′-GAUCACCUCCUUUCUA-3′. This finding further suggests that both CCUCC and downstream bases are conserved regions that are preferred in SD:aSD pairing in most species.

To offer a plausible reason for the unexpected length of the 3′ TAIL in *L. interrogans*, it is worth noting that the dependence on the SD:aSD interaction for efficient translation is dynamic (Nakagawa *et al.* 2017). In genes that have strong secondary structure within the TIR, ribosome recruitment is facilitated by RPS1 (Nakagawa *et al.* 2010; Osterman *et al.* 2013). This protein binds U-rich regions (Boni *et al.* 1991; Komarova *et al.* 2005) to unfold double-stranded RNA (Qu *et al.* 2012; Duval *et al.* 2013). Furthermore, RPS1’s domains appear to be under higher functional constraint in species possessing few SD-containing genes, such as *L. interrogans* (Nakagawa *et al.* 2010, 2017); the reliance on RPS1 reduces the dependence on a SD:aSD interaction and may relax 3′ TAIL boundary constraints.

Notably, four species (*D. deserti*, *M. pneumoniae*, *L. pneumophila*, and *L. interrogans*) have a secondary peak of mapped reads within 20 nt downstream of CCUCC (Figure 1, Table 1, Supplementary Fig. S1). We propose that the secondary peak farther downstream is the pre-16S rRNA; it is too far downstream of CCUCC to be considered as the mature 16S rRNA 3′ end based on sequence conservation (Nakagawa *et al.* 2010). The prominence of this second peak may be due to the accumulation of the endoribonuclease cleaved pre-16S rRNA intermediate, because the localization of exoribonuclease to this precursor sequence is a rate limiting step. However, the intermediate sequence is rapidly continuously degraded once it is targeted by these enzymes. This would explain the lack of sequences mapped between the mature 16S rRNA and the intermediate sequence (the two peaks) (Figure 1c, Supplementary Fig. S1).

### The 3′ TAIL terminal bases are preferred in SD:aSD binding

We define an aSD site to be preferred if the observed number of times the base is involved in SD pairing is greater than expected. In the absence of SD usage bias, a putative SD sequence of 4 to 12 nt can be expected to pair anywhere within the boundary of the aSD sequence, as long as complete complementarity is achieved. Here, we designate the aSD sequence to constitute the 3′ TAIL, beginning with the conserved 5′-GAUCA-3′, followed by the core motif CCUCC, and extended by variable lengths of terminal bases characterized herein (Table 1, *e.g.*, in *L. monocytogenes*, the aSD sequence is 5′GAUCACCUCCUUUCU-3′ and the terminal bases are 5′-UUUCU-3′). Then, taking *L. monocytogenes* as example, the maximum number of possible pairs at the first complementary aSD site (*aSD_1*) by the total pool of 4 nt to 12 nt putative SD sequences is calculated by equation (1), with N_m_ denoting N number of putative SD sequences of length m:

$$aSD\_1=\overset{12}{\underset{m=4}{\sum }}\frac{N_{m}}{15-m+1}\;$$ (1)

However, the number of possible base-pairs resulting in perfect complementarity varies. For example, a 12 nt putative SD sequence may start pairing at the first, but not the sixth, base on a complementary aSD sequence that is 15 nt long, and the maximum usage of the sixth aSD site (*aSD_6*) is calculated instead by equation (2):

$$aSD\_6=4\times \frac{N_{4}}{12}+5\times \frac{N_{5}}{11}+6\times \frac{N_{6}}{10}+6\times \frac{N_{7}}{9}+6\times \frac{N_{8}}{8}+6\times \frac{N_{9}}{7}+6\times \frac{N_{10}}{6}+6\times \frac{N_{11}}{5}+5\times \frac{N_{12}}{4}$$ (2)

The expected usage is then calculated by taking the relative proportions of maximum usage at each site (adding up to 1) multiplied by the total number of observed putative SD sequences of various lengths. These calculations are implemented in DAMBE (Xia 2018b) under the ‘Analyze 5UTR’ function.

**Figure 2:**
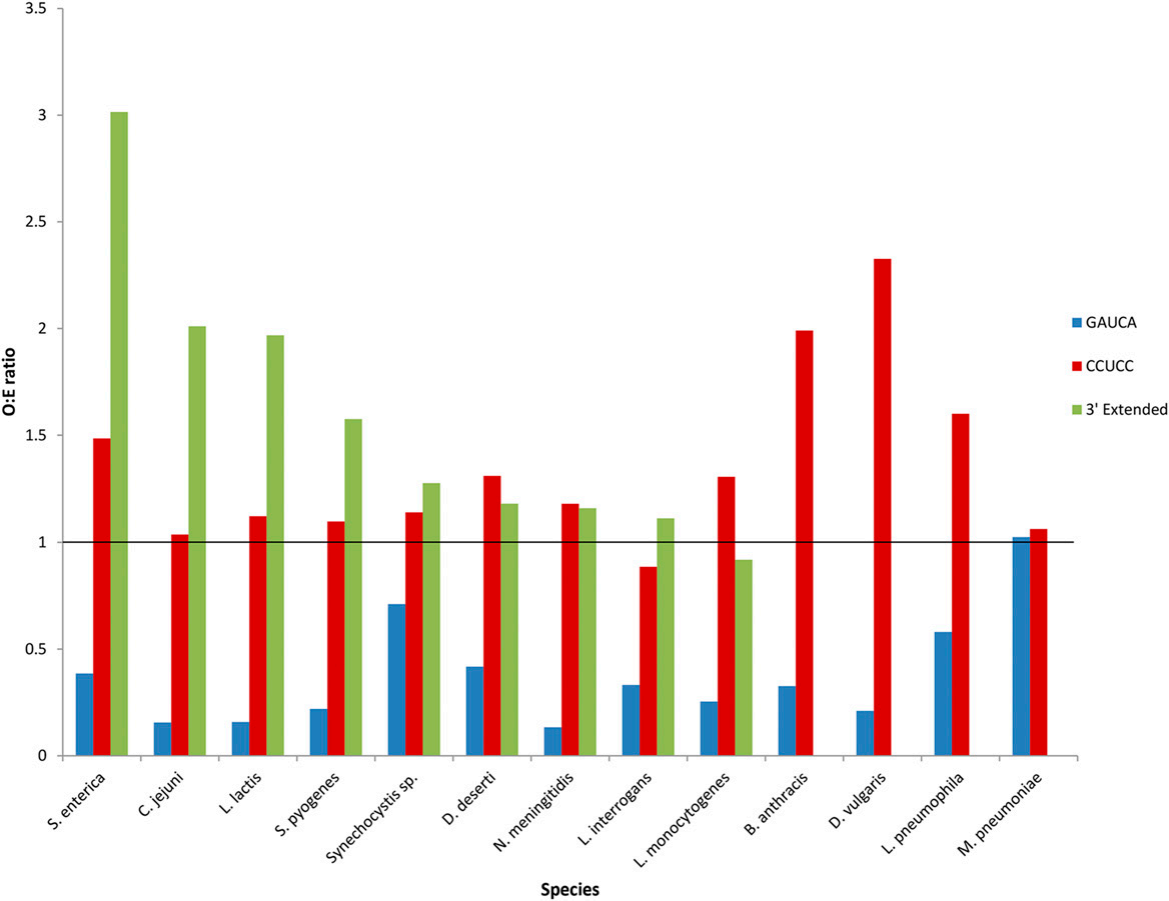
Mean ratio of observed over expected SD:aSD complementarity (O:E ratio) in 13 species at conserved 5′-GAUCA-3′ (blue), and 5′-CCUCC-3′ motifs (red). The average O:E ratio is also shown for the characterized sequences downstream of CCUCC (green) in the nine bacterial species that have extended ends.

As defined, a preferred aSD site will have an observed to expected SD:aSD usage ratio (O:E) > 1. Since expected SD:aSD count is calculated in absence of any selection bias in SD usage, an O:E > 1 suggests presence of selection bias in observed SD usage. Figure 2 shows an average O:E > 1 at CCUCC for all species, with the exception of *L. interrogans*. Indeed, CCUCC is preferentially used in SD:aSD pairing. Meanwhile, average O:E is <1 for 5′-GAUCA-3′ in all species except *M. pneumoniae*; hence, this conserved region is avoided by SD:aSD pairing in most species. Lastly, in keeping with expectations, conserved regions downstream of CCUCC have an O:E > 1 in all species except *L. monocytogenes*. These observations indicate that downstream bases are retained because they are preferred in SD:aSD binding, despite their weaker binding affinity than CCUCC. This result corroborates recent studies suggesting that intermediate SD:aSD complementarity increase initiation efficiency (Osterman *et al.* 2013; Hockenberry *et al.* 2017; Wei *et al.* 2017).

In this study we present an RNA-Seq based approach to characterize the 3′ end of mature 16S rRNA in bacterial species across different lineages. There is weaker 3′ TAIL conservation at the RNA level than at the DNA level. Nonetheless, the presence of 3′ termini bases downstream of CCUCC falls within the conserved boundary at the genomic level. Furthermore, the usage of terminal bases is favored in SD:aSD binding. Alternatively, RPS1-mediated initiation may relax the functional constraint at the 3′ TAIL of *L. interrogans*, explaining its exceptional length. Our findings complement previous studies investigating the role of CCUCC in translation initiation and suggest that transcribed bases downstream of this canonical motif also play an important role in translation efficiency.
